# Assessment of Airflow and Oximetry Signals to Detect Pediatric Sleep Apnea-Hypopnea Syndrome Using AdaBoost

**DOI:** 10.3390/e22060670

**Published:** 2020-06-17

**Authors:** Jorge Jiménez-García, Gonzalo C. Gutiérrez-Tobal, María García, Leila Kheirandish-Gozal, Adrián Martín-Montero, Daniel Álvarez, Félix del Campo, David Gozal, Roberto Hornero

**Affiliations:** 1 Biomedical Engineering Group, University of Valladolid, 47011 Valladolid, Spain; gonzalo.gutierrez@gib.tel.uva.es (G.C.G.-T.); maria.garcia@tel.uva.es (M.G.); adrian.martin@gib.tel.uva.es (A.M.-M.); dalvarezgo@saludcastillayleon.es (D.Á.); fsas@telefonica.net (F.d.C.); robhor@tel.uva.es (R.H.); 2 Centro de Investigación Biomédica en Red en Bioingeniería, Biomateriales y Nanomedicina (CIBER-BBN), Instituto de Salud Carlos III, 28029 Madrid, Spain; 3 Department of Child Health, The University of Missouri School of Medicine, Columbia, MO 65212, USA; gozall@health.missouri.edu (L.K.-G.); gozald@health.missouri.edu (D.G.); 4 Sleep-Ventilation Unit, Pneumology Service, Río Hortega University Hospital, 47012 Valladolid, Spain

**Keywords:** sleep apnea–hypopnea syndrome, airflow, oximetry, AdaBoost, spectral analysis, nonlinear analysis

## Abstract

The reference standard to diagnose pediatric Obstructive Sleep Apnea (OSA) syndrome is an overnight polysomnographic evaluation. When polysomnography is either unavailable or has limited availability, OSA screening may comprise the automatic analysis of a minimum number of signals. The primary objective of this study was to evaluate the complementarity of airflow (AF) and oximetry (SpO_2_) signals to automatically detect pediatric OSA. Additionally, a secondary goal was to assess the utility of a multiclass AdaBoost classifier to predict OSA severity in children. We extracted the same features from AF and SpO_2_ signals from 974 pediatric subjects. We also obtained the 3% Oxygen Desaturation Index (ODI) as a common clinically used variable. Then, feature selection was conducted using the Fast Correlation-Based Filter method and AdaBoost classifiers were evaluated. Models combining ODI 3% and AF features outperformed the diagnostic performance of each signal alone, reaching 0.39 Cohens’s kappa in the four-class classification task. OSA vs. No OSA accuracies reached 81.28%, 82.05% and 90.26% in the apnea–hypopnea index cutoffs 1, 5 and 10 events/h, respectively. The most relevant information from SpO_2_ was redundant with ODI 3%, and AF was complementary to them. Thus, the joint analysis of AF and SpO_2_ enhanced the diagnostic performance of each signal alone using AdaBoost, thereby enabling a potential screening alternative for OSA in children.

## 1. Introduction

Childhood Obstructive Sleep Apnea (OSA) syndrome is a sleep disorder in which airflow is intermittently interrupted or decreased during sleep, mainly due to the obstruction of the upper airway [1,2]. Events of absence (apnea) or reduction (hypopnea) in air exchange caused by these obstructions reduce the oxygenation of blood, and disturb the normal progression of sleep stages, which leads to restless sleep, daytime sleepiness and behavioral problems [1,2]. Untreated pediatric OSA can lead to neurocognitive deficits, cardiovascular complications and negative consequences related to development and behavior [3,4,5]. OSA prevalence in children is between 1–5%, with a high proportion of cases likely undiagnosed [2,3]. The gold standard of OSA diagnosis is a nocturnal in-lab polysomnography (PSG) [2,3,6], which consists in the analysis of children’s sleep by recording an extensive number of biomedical signals [6,7,8]. Physicians then calculate the apnea–hypopnea index (AHI), the rate of apneas and hypopneas per hour of sleep (e/h). Pediatric OSA severity is then classified into three levels: Mild (1 ≤ AHI < 5 e/h), Moderate (5 ≤ AHI < 10 e/h) or Severe (AHI ≥ 10 e/h) [9]. Early and timely diagnosis of OSA is of crucial importance to allow children to be assessed for surgical treatment, depending on their severity and comorbidities, while also preventing the negative consequences of the disease [9].

Costs, complexity and scarce availability of pediatric sleep laboratory facilities are the main disadvantages of PSG [8,10]. Several alternatives have been proposed to overcome them. Mostly accepted approaches aim to reduce the complexity of PSG, and usually rely on the analysis of a reduced set of signals. Some examples include nocturnal oximetry and respiratory polygraphy, which includes the recording of airflow (AF) and oximetry (SpO_2_) signals [7,10,11,12,13]. AF reflects the respiratory activity during sleep, while SpO_2_ represents the arterial oxygen saturation [14]. Both signals are involved in the definition of apnea or hypopnea events and, therefore, provide useful information to detect OSA [3,14]. Thus, it is possible to retrieve most of the information related to apneas and hypopneas using the AF and SpO_2_ signals included in the PSG [8,12]. Some studies have reported that oximetry alone is less reliable than respiratory polygraphy [7,13]. Hence, the joint analysis of AF and SpO_2_ from PSG can enhance pediatric OSA screening with respect to using SpO_2_ only.

Previous studies focused on the assessment of AF or SpO_2_ signals as simplified alternatives to PSG [15,16,17]. They involved adult [18,19,20,21,22] and pediatric [23,24,25,26,27,28,29,30,31,32] subjects as well as different automated signal processing methods. Regarding automatic analysis of SpO_2_ signals, several methods have been proposed to detect OSA in children. These studies comprised time and frequency domain analyses [23,24,25,26], as well as nonlinear methods and various oximetric indexes [25,26,27,28,29]. Studies involving AF signals comprised spectral analysis [30], irregularity and variability analyses [31], and nonlinear recurrence plots [32]. Some of these studies established the complementarity of AF with the 3% oxygen desaturation index (ODI) [30,32]. This clinical variable from SpO_2_ counts the number of oxygen desaturations >3% per hour of recording, and it is used as simplified screening test in clinical practice due to pediatric sleep laboratories are not widely available to perform complete PSG in children [12,13]. Both ODI 3% and ODI 4% have been proposed in conjunction with other oximetric variables in the screening of OSA using nocturnal oximetry [12]. However, there is a scarcity of studies regarding the joint analysis of AF and SpO_2_ signals in children [33]. Only one recent study in adults focused on a direct comparison of the diagnostic ability of AF and SpO_2_ signals, which evidenced their complementarity [34]. Pediatric OSA differs from the adult disease in the symptom spectrum, negative outcomes and the PSG findings, such that adult criteria cannot be used for diagnosis in children [1,3,5,9,35]. Accordingly, the definition of apneic events and the AHI cutoffs used to define OSA severity are different in children [6,14,17]. Here, we propose to evaluate the diagnostic ability of AF and SpO_2_ signals using a pediatric population.

From a machine learning point of view, previous studies focused on the automatic detection of pediatric OSA using simple and widespread algorithms. These approaches included Logistic Regression (LR) [23,24,25,27,28,30,31,36,37], Linear or Quadratic Discriminant Analysis (LDA, QDA) [38,39,40], and Support Vector Machines (SVM) [41]. Other recent studies relied on more complex Multilayer Perceptron (MLP) neural networks [26,29,32,42,43]. Most of these algorithms can be hardly generalizable due to their simplicity and susceptibility to overfitting the training data [44]. However, ensemble learning approaches have not yet been evaluated despite their high performance in studies involving AF or SpO_2_ recordings from adults [19,22,45]. To avoid this limitation, we propose the use of AdaBoost, a well-known generalizable ensemble learning algorithm [46].

The main hypothesis of this study is that an adequate combination of the information from AF and SpO_2_ signals can yield higher diagnostic performance than each of these signals separately. Therefore, our primary goal is to compare the information of AF and SpO_2_ signals to detect OSA in children and evaluate their complementarity. Additionally, the secondary objective is to evaluate the diagnostic ability of AdaBoost classifiers using features from each signal separately and combined.

## 2. Database

The database used in this study comprised 974 pediatric subjects referred to the Comer Children’s Hospital, University of Chicago Medicine (Chicago, IL, USA), with clinical suspicion of OSA. This study was conducted according to the Declaration of Helsinki. The legal caretakers of each subject provided the informed consent and the Ethics Committee of the University of Chicago Medicine approved the study protocol (#11-0268-AM017, # 09-115-B-AM031, and # IRB14-1241). In-laboratory sleep studies were performed with a digital polysomnography device (Nihon Kohden America Inc., CA, USA). Subjects were evaluated according to the rules defined by the American Academy of Sleep Medicine (AASM) [14], including the computation of the AHI. The subjects of the database were classified according to OSA severity in four groups: No OSA (AHI < 1 e/h), Mild OSA (1 ≤ AHI < 5 e/h), Moderate OSA (5 ≤ AHI < 10 e/h) and Severe OSA (AHI ≥ 10 e/h). These severity groups were chosen in accordance with previous studies [8,9,26]. Table 1 summarizes sociodemographic—Age, number of males and females—And clinical data—Normalized body mass index (BMI *z*-score), AHI, number of patients with OSA—Of the subjects involved in this study. They were randomly split into a training set (60%) and a test set (40%). No significant differences were found in age, sex, BMI *z*-score and AHI between the two sets (*p* > 0.01, Mann–Whitney *U* test). The training set was used to fix the optimum values of the method parameters using a bootstrap approach and train the classifiers. The test set was used to evaluate the diagnostic performance of our algorithm.

## 3. Methods

**Figure 1:**
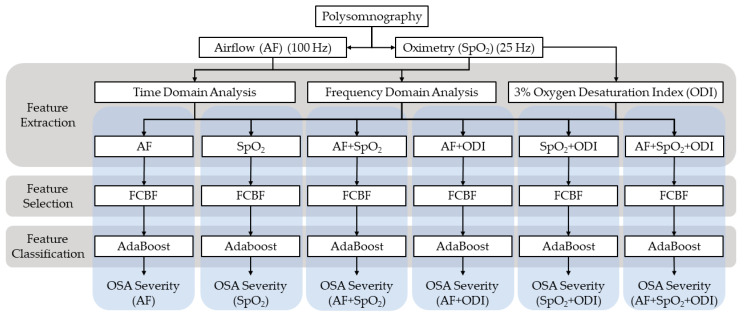
Workflow of the proposed methodology. Fast Correlation-Based Filter (FCBF); Obstructive Sleep Apnea (OSA).

In this study, AF and SpO_2_ signals extracted from 974 PSG recordings were analyzed. AF signals were sampled at *f_s_* = 100 Hz, while SpO_2_ signals were obtained from the pulse oximeter at *f_s_* = 25 Hz, as recommended by the AASM [14]. Figure 1 shows the workflow of the proposed methodology. After preprocessing, features were extracted using time and frequency-based analyses. This study was intended to assess the complementarity of the features extracted from AF and SpO_2_ signals, and therefore different feature sets were assessed: AF-derived features, SpO_2_-derived features and both AF and SpO_2_ features. We also split the experiments in two situations: with and without ODI 3%. Six settings were thus investigated, namely: ‘AF’, ‘SpO_2_′, ‘AF + SpO_2_′, ‘AF + ODI’, ‘SpO_2_ + ODI’ and ‘AF + SpO_2_ + ODI’. Feature selection was conducted in these feature sets independently to establish optimum subsets of features before the classification stage. Finally, the selected features were used to train and evaluate six independent AdaBoost classifiers.

### 3.1. Preprocessing

AF and SpO_2_ signals were preprocessed in order to remove artifacts and signal loss intervals, as well as to normalize the amplitude values. In the case of SpO_2_ signals, samples with values lower than 50% of saturation and intervals with abrupt changes of oxygen saturation greater than 4% per second were removed [26,29]. AF signals were filtered using a low-pass filter (cutoff frequency of 1.5 Hz) and subsequently normalized [31,32]. Artifacts in the AF signal were removed using a method based on the standard deviation and the kurtosis of 30 s segments, as in previous studies [32].

### 3.2. Feature Extraction: Time and Frequency Domain Analyses

The feature extraction stage comprised the characterization of AF and SpO_2_ signals using automatic signal processing algorithms. In this study, analyses were performed in time and frequency domains. Extracted features summarize the information about the alterations of the signals properties and the recurrence of apneic events, and have been widely assessed in previous studies dealing with automatic detection of adult and pediatric OSA [19,20,26,31].

#### 3.2.1. Time-Domain Moments and Nonlinear Analysis

Five time domain statistical moments were computed: mean (M1T), standard deviation (M2T), skewness (M3T), kurtosis (M4T) and median (MedT) [20,26]. Besides, three nonlinear features have also been obtained: Central Tendency Measure (CTM) [47], Lempel–Ziv Complexity (*LZC*) [48] and Sample Entropy (*SampEn*) [49]. Signals were segmented into epochs of 30 s prior to perform time-domain and nonlinear analyses, and each feature was calculated as the average value across all segments.

CTM is a measure of the variability of a signal [47]. It is based on plots of first order differences: given a signal *x*(*n*) of length *N*, the values *x*(*n* + 2) − *x*(*n* + 1) are represented against *x*(*n* + 1) − *x*(*n*) in a scatter plot. CTM is the rate of differences that lie inside a circle of fixed radius *r* [20,47]:

(1) $$CTM=\frac{\sum_{i=1}^{N-2}\delta \left(i\right)}{N-2};\;\delta \left(i\right)=\{\begin{array}{l} 1\;if\;\sqrt{{\left[x\left(i+2\right)-x\left(i+1\right)\right]}^{2}+{\left[x\left(i+1\right)-x\left(i\right)\right]}^{2}}<r\\ 0\;otherwise \end{array}.$$

The computation of CTM relies on the parameter *r*. The values of *r* were independently set for AF and SpO_2_ signals by maximizing the absolute value of the Spearman’s correlation coefficient (*ρ*) between CTM and the AHI in the training set [26,31].

LZC is a nonparametric measure of the complexity of a time series. The LZC of a sequence increases as more subsequences are contained in it. To analyze the subsequences, the signal is converted to binary by applying a threshold, usually the median value of the samples [20,26,48]. Then, the binary data is scanned and a counter *c*(*n*) is increased as more different sequences are found in the data. LZC is the coefficient [20,48]:

(2) $$LZC=\frac{c\left(n\right)}{b\left(n\right)};b\left(n\right)=\frac{n}{\log_{2}n}.$$

In (2), the normalizing factor *b*(*n*) is equal to the theoretical upper bound of *c*(*n*). [48].

*SampEn* is a statistic used to measure irregularity in biomedical signals [49]. It has been widely employed to characterize fluctuations of AF and SpO_2_ signals [18,20,27,49]. Given a signal of length *N*, *SampEn* is defined as the negative logarithm of the conditional probability of two similar sequences of length *m* remaining similar (distance lower than *r*) after the length of the sequence increases in one sample (length *m* + 1) [20,49]:

(3) $$SampEn\left(m,r,N\right)=-\log[\frac{A^{m}\left(r\right)}{B^{m}\left(r\right)}],$$

where *A^m^*(*r*) and *B^m^*(*r*) are the average number of similar sequences of length *m* + 1 and *m*, respectively. For each signal, we fixed the optimum values of parameters *m* and *r* to those which maximized the absolute value of Spearman’s *ρ* of *SampEn* with the AHI in the training set [26,31]. The trials were set with *m* in the range 1–3 and *r* in the range 0.05–0.3 times the standard deviation of the signal [50].

#### 3.2.2. Spectral Analysis

Frequency domain features were obtained after the estimation of the Power Spectral Density (PSD) of the signals using the non-parametric Welch method [51]. The signals were segmented in epochs with 50% overlap using a Hamming window of 2^14^ and 2^16^ samples for SpO_2_ and AF, respectively. Window lengths were defined as the minimum power of two that encompasses a segment duration greater than 10 min, a tradeoff between spectral resolution and number of segments [51]. The PSD estimation was then obtained by averaging the PSDs of segments [51].

**Figure 2:**
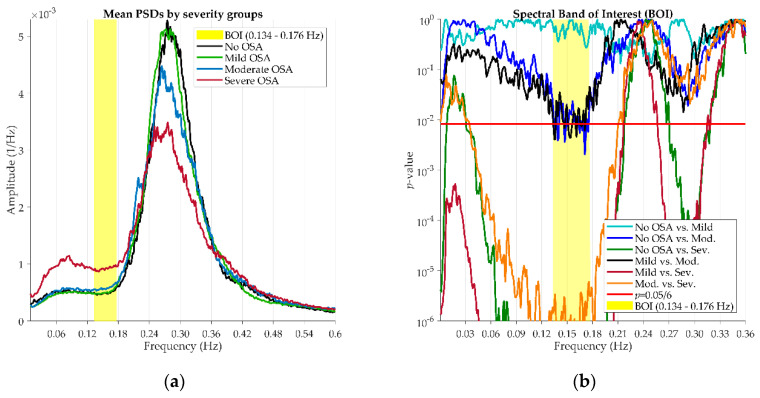
(**a**) Mean Power Spectral Densities of AF signals for each Obstructive Sleep Apnea (OSA) severity group. (**b**) Definition of the spectral Band of Interest (BOI).

Once the signals PSDs were estimated, we defined the spectral band of interest (BOI) of the AF signal as the band where the amplitude of the PSD of AF differs among severity groups. In the case of SpO_2_ signal, we employed the spectral BOI between 0.020–0.044 Hz defined in previous studies [26]. Following the same methodology, we sought a spectral BOI for the AF signal. The Mann–Whitney *U* test was used to compare the values of the PSD and find frequency ranges showing the highest statistically significant differences among OSA severity groups [18,26,33]. Figure 2a shows the average PSDs of No OSA, Mild, Moderate and Severe OSA subjects in the training set. The *p*-values obtained for each frequency are shown in Figure 2b. Only frequencies between 0–0.36 Hz are displayed to allow a proper visualization of the BOI. As four severity groups were involved in the comparison, a total of six pairwise comparisons were conducted. A spectral BOI was found in 0.134–0.176 Hz, where the maximum number of comparisons showed statistically significant differences (*p* < 0.05/6, Bonferroni correction).

Seven features were obtained from the PSD values in the spectral BOI [26,30]: first to fourth statistical moments (M1F–M4F), median (MedF), maximum (MaxF) and minimum (MinF). Additionally, the full spectrum of the signals was characterized by obtaining four features: the median frequency (FreqM), the spectral entropy (SpecEn) and the quadratic and cubic spectral entropies (SpecEn^2^ and SpecEn^3^, respectively) [20,31]. FreqM is defined as the frequency (*f*) that accomplishes that 50% of the total power is below that frequency.

(4) $$\sum_{f=0}^{f=FreqM}PSD\left(f\right)=\frac{1}{2}\sum_{f=0}^{f=f_{s}/2}PSD\left(f\right).$$

SpecEn^i^ is defined as the Shannon entropy of the frequency distribution provided by the normalized *i*-th power of the PSD (*PSD^i^_n_*) [31]:

(5) $$SpecEn^{i}=-\frac{1}{\log N}\sum_{f=0}^{f=f_{s}/2}PSD^{i}{}_{n}\left(f\right)\cdot \log\left[PSD^{i}{}_{n}\left(f\right)\right]\left(i=1,2,3\right),$$

where *N* is the number of samples of *PSD_n_* in 0 − *f_s_*/2. SpecEn indirectly estimates the irregularity of a signal since higher SpecEn values are expected from flatter PSDs, with no dominant frequencies [31].

#### 3.2.3. Oxygen Desaturation Index

Finally, ODI 3% was computed from the SpO_2_ signal as the number of desaturations greater than or equal to 3% from the baseline per hour of recording. This oximetric index has been found useful in previous approaches focused on the detection of childhood OSA [25,26,27,34].

### 3.3. Feature Selection: Fast Correlation-Based Filter

In the feature extraction stage, 19 signal processing-derived features were extracted for each signal: five time-domain statistics, three nonlinear measures, seven statistics from the BOI and four from the full spectrum measures. ODI 3% was added to these 38 features, so the total number of features was 39. Feature selection was implemented in this study to identify relevant and complementary features of AF and SpO_2_ signals and derive simpler models with reduced chances of overfitting [52]. We employed the Fast Correlation-Based Filter (FCBF) method [53] prior to the feature classification stage. This classifier-independent method identifies the most relevant features and removes redundant ones to obtain the optimum subset of features [53]. FCBF is based on measures of the symmetrical uncertainty (*SU*) between features *X_i_* and *X_j_*. It is defined as [53]:

(6) $$SU\left(X_{i}|X_{j}\right)=2\frac{H\left(X_{i}\right)-H(X_{i}|X_{j})}{H\left(X_{i}\right)+H\left(X_{j}\right)},$$

where *H*(*X_i_*) is the Shannon entropy of *X_i_*, and *H*(*X_i_*|*X_j_*) is the Shannon entropy of the feature *X_i_* when *X_j_* is observed. The relevance of *X_i_* is defined as *SU* (*X_i_*|*Y*)—Being *Y* the AHI—And redundancy is defined as *SU*(*X_i_*|*X_j_*). The criteria to remove *X_i_* is the following [53]:

(7) $$SU\left(X_{j}|Y\right)\geq SU\left(X_{i}|Y\right),\;\mathrm{and}\;SU\left(X_{i}|X_{j}\right)\geq SU\left(X_{i}|Y\right).$$

FCBF was combined with bootstrapping to reduce dependency on the training data and improve generalization [44,52]. We obtained 1000 bootstrap replicates from the training data and the FCBF algorithm was applied to each one [26,29,44]. Features selected at least 500 times formed the optimum subset of features [26,29,52].

### 3.4. Classification: Multiclass AdaBoost

The classification stage was aimed at predicting the severity of OSA using the features selected in the previous stage. As we described in Section 2, subjects were classified in four groups according to the severity of OSA. We employed the multiclass AdaBoost classifier, an ensemble learning method based on boosting [44,46]. The main idea behind ensembles is to combine several classifiers to build a robust one with an increased generalization ability [44]. Base classifiers used to construct ensembles are usually weak and simple decision rules, such as decision trees or LDA classifiers [46]. The crucial rule of ensembles is diversity, that is, weak classifiers need to be trained with different representations of the training set [46]. This way, each weak classifier becomes an expert in a certain area of the feature space and the ensemble makes its predictions based on a committee of diverse and complementary classifiers [46]. Boosting methods are ensembles characterized by sequential training of base classifiers. At each iteration, a new base classifier is trained giving higher weights to instances in which the previous base classifier failed to make a prediction. After a sufficient number of base classifiers are trained, final predictions are obtained by weighted vote of base classifiers [46].

AdaBoost is the most widespread boosting method [46]. In this study, we employed the algorithm AdaBoost.M2, which allows multiclass classification [54]. We used LDA as base classifier since it was proven useful in previous OSA-related studies [19,22]. The training data comprised *N* feature vectors, ***x_i_***, with labels, *y_i_* (*i* = 1, …, *N*). The number of iterations, *L*, was experimentally tuned. For each iteration *t* (*t* = 1, …, *L*), a single base classifier is trained using a version of the training data with weights *w_i_*(*t*). First, the distribution *D_t_*(*i*) is calculated as [54]:

(8) $$D_{t}\left(i\right)=\frac{W_{i}^{t}}{\sum_{i=1}^{N}W_{i}^{t}}\;,$$

where with *W_i_*:

(9) $$W_{i}^{t}=\sum_{y\neq y_{i}}w_{i,y}^{t}.$$

The base classifier is then trained with the distribution *D_t_*(*i*). The trained base classifier generates a weak prediction *h_t_*(*x*,*y*) and the pseudo-loss *ε_t_* is calculated [54]:

(10) $$\varepsilon_{t}=\frac{1}{2}\sum_{i=1}^{N}D_{t}\left(i\right)\left[1-h_{t}\left(x_{i},y_{i}\right)+\sum_{y\neq y_{i}}\frac{w_{i,y}^{t}}{W_{i}^{t}}h_{t}\left(x_{i},y\right)\right].$$

Then, the weight update coefficient of base classifier *t*, *β_t_*, is obtained [54]:

(11) $$\beta_{t}=\frac{\varepsilon_{t}}{1-\varepsilon_{t}}.$$

Additional regularization was added using a modified *β_t_* with a learning rate parameter *ν*:

(12) $$\beta_{t}={\left(\frac{\varepsilon_{t}}{1-\varepsilon_{t}}\right)}^{\nu },$$

where with *ν* in the range 0–1. Then, *w_i_* of the instances ***x_i_*** for the next iteration *t* + 1 are computed as [54]:

(13) $$w_{i,y}^{t+1}=w_{i,y}^{t}\beta_{t}^{\frac{1}{2}\left(1+h_{t}\left(x_{i},y_{i}\right)-h_{t}\left(x_{i},y\right)\right)}.$$

The final prediction of AdaBoost *H*(*x*) is obtained by means of weighted vote [54]:

(14) $$H\left(x\right)=\arg\underset{y}{\;\max}\sum_{t=1}^{L}\left(\log\frac{1}{\beta_{t}}\right)h_{t}\left(x,y\right).$$

### 3.5. Model Optimization and Training

Our database was split into a training set and a test set. The training set was used to derive the optimal number of iterations (*L*) and the learning rate (*ν*) of the AdaBoost algorithm, while the test set was intended to evaluate the models in new data. The hyperparameters *L* and *ν* are involved in the number of base classifiers to be trained and the calculation of the weight update coefficient *β_t_*, respectively. We set trials to estimate the performance of AdaBoost in the training set using the Cohen’s Kappa (*κ*) [55], with *L* in the range between two and 10,000 classifiers and *ν* in the range 0.1–1. Cohen’s *κ* is less sensitive to class imbalance in comparison with the error rate [55]. We used the 0.632 bootstrap validation method to estimate *κ* with reduced chances of overfitting [44]. We obtained 1000 new bootstrap replicates from the training data and trained a model for each one. Repeated instances are frequent in a bootstrap replicate, whereas other instances are not selected [44]. Unselected instances formed a validation set used to evaluate the trained model. The estimate of *κ* using 0.632 bootstrap, *κ_B_*(*i*) (*i* = 1, …, 1000), is [44]:

(15) $$\kappa_{B}\left(i\right)=0.632\cdot \kappa_{BValidation}\left(i\right)+0.368\cdot \kappa_{BTraining}\left(i\right),$$

where *κ_BValidation_*(*i*) and *κ_BTraining_*(*i*) are the values of *κ* obtained when the model is evaluated in the validation and training bootstrap datasets, respectively [44]. The final *κ_B_* is the average of *κ_B_*(*i*) over *i* [44]. AdaBoost classifiers were trained using the overall training set with optimum *L* and *ν* fixed.

### 3.6. Statistical Analysis

A correlation analysis was conducted in the training set to evaluate the relationship between extracted features and the AHI using the Spearman’s *ρ*. Statistically significant differences between severity groups were also examined in the training set using the Kruskal–Wallis test (*p* < 0.01/6, Bonferroni correction), since features did not pass the Lilliefors normality test. Results obtained in the test set were summarized in a four-class confusion matrix. The agreement between the predicted severity and the gold standard was assessed using the four-class accuracy (*Acc_4_*) and *κ*. Diagnostic ability in the common AHI cutoffs was evaluated using Sensitivity (Se, percentage of diseased subjects correctly classified), Specificity (Sp, percentage of healthy subjects correctly classified), Accuracy (Acc, percentage of subjects correctly classified), Positive and Negative Predictive Value (PPV, NPV, percentage of subjects correctly classified as positives/negatives) and Positive and Negative Likelihood Ratios (LR+ = Se/(1 − Sp), LR− = (1 − Se)/Sp).

## 4. Results

### 4.1. Preprocessing. Parameters Optimization in the Training Set

**Figure 3:**
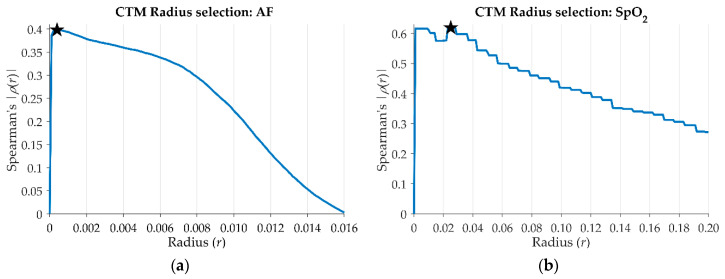
Absolute value of Spearman’s correlation coefficient (*ρ*) between Central Tendency Measure (CTM) and the apnea–hypopnea index as a function of radius (*r*) in the training set. (**a**) Airflow (AF) signal; (**b**) oximetry (SpO_2_) signal.

Artifacts in both AF and SpO_2_ signals were removed in the preprocessing stage. The rates of rejected data—Median [interquartile range]—Were 5.65% [1.76%, 10.30%] and 5.36% [1.51%, 9.17%] of the total recording time for AF and SpO_2_ signals, respectively. The amount of discarded data was low comparing to the length of the overnight recordings and both signals were similarly affected by artifacts (*ρ* = 0.5394, Spearman’s rank correlation). In addition, no substantial differences were found between the rates of rejected data of AF and SpO_2_ (*p* = 0.4175, Wilcoxon signed rank test). Figure 3 shows the absolute value of the Spearman’s *ρ* of CTM with AHI for varying *r* in the training set. The maximum values of |*ρ*(*r*)| were reached using *r* = 0.0004 in AF and *r* = 0.025 in SpO_2_. Following the same criteria for SampEn, the optimum parameters were *m* = 2 and *r* = 0.05 for AF, and *m* = 3 and *r* = 0.05 for SpO_2_ (Table 2).

### 4.2. Statistical Analysis in the Training Set: Individual Features

Table 3 summarizes the results of the correlation and statistical differences between severity groups in the training set. Several features extracted from both signals showed significant differences between severity groups. Two nonlinear features from AF as well as some time and frequency-domain measures from both signals showed no statistically significant differences. These features were generally associated with the lowest correlations obtained in this study. In general, SpO_2_ features obtained the highest correlations with the AHI, whereas correlations of several AF features were weaker but significant. It is also remarkable that both time and frequency domain analyses showed statistically significant correlations with the AHI. CTM obtained the highest correlation among AF features and the highest correlations among nonlinear features in both signals. Regarding the spectral analysis-derived features, correlations were also higher in the SpO_2_ signal. Nevertheless, SpecEn-derived features showed higher correlations when they were applied in the AF signal in comparison with SpO_2_. Overall, ODI 3% achieved the highest correlation with the AHI.

### 4.3. Feature Selection in the Training Set

**Figure 4:**
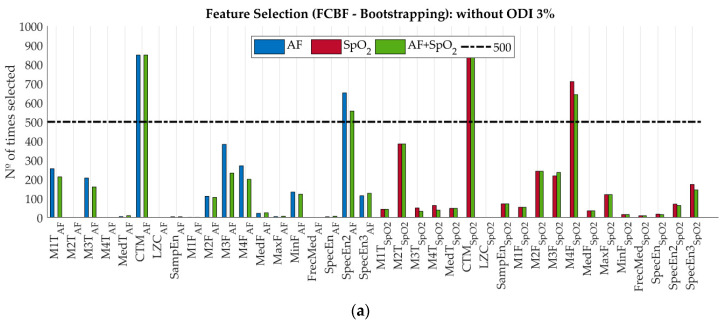

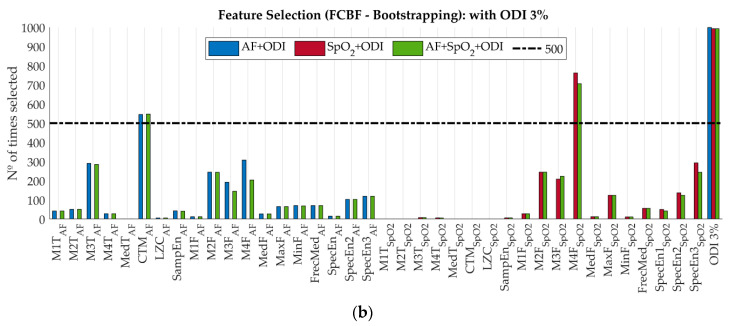
Results of the feature selection in the training set using Fast Correlation-Based Filter (FCBF). (**a**) sets without ODI 3%; (**b**) sets with ODI 3%.

Figure 4 shows the histograms of the number of times each feature was selected using different groups of features in the training set: ‘AF’, ‘SpO_2_’, ‘AF + SpO_2_’, ‘AF + ODI’, ‘SpO_2_ + ODI’ and ‘AF + SpO_2_ + ODI’. Results of feature selection without ODI 3% are shown in Figure 4a. Selected features from the ‘AF’ (CTM_AF_, SpecEn^2^_AF_) and the ‘SpO2’ (CTM_SpO2_, M4F_SpO2_) sets were selected again using the ‘AF + SpO_2_’ set. In this case, no redundant features were found when both signals were combined. Results with ODI 3% are shown in Figure 4b. In these three cases, ODI 3% was found to be the most relevant feature and made SpecEn^2^_AF_ and CTM_SpO2_ redundant. Furthermore, CTM_AF_, and M4F_SpO2_ were nonredundant.

### 4.4. Model Optimization in the Training Set

**Figure 5:**
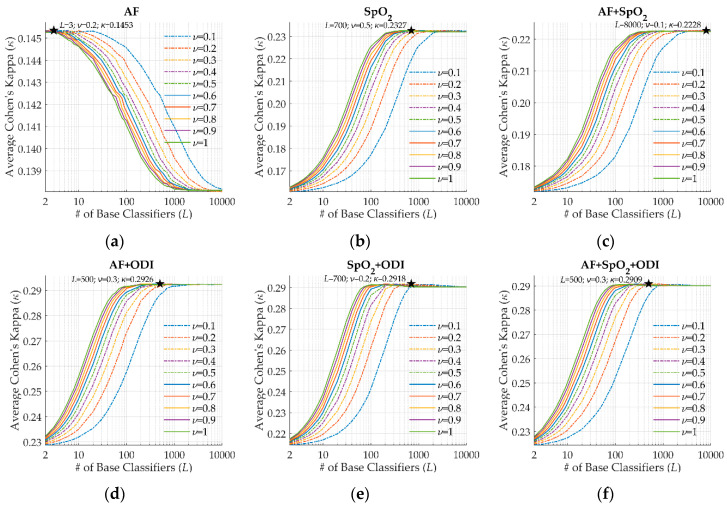
Performance in the training set of AdaBoost models as a function of the number of base classifiers (*L*) and the learning rate (*ν*), with their optimum values highlighted. (**a**) AF subset; (**b**) SpO_2_ subset; (**c**) AF + SpO_2_ subset; (**d**) AF + ODI subset; (**e**) SpO_2_ + ODI subset; (**f**) AF + SpO_2_ + ODI subset.

We trained an independent AdaBoost ensemble model for each of the six subsets of selected features using training data. Hence, different optimum values of *L* and *ν* were obtained in each case to optimize the performance. Figure 5 shows the bootstrap estimate of *κ* in the training set for the corresponding trials. AdaBoost models trained with features from the AF signal did not yield higher *κ* as *L* increased, as shown in Figure 5a. In this case, a large value of *L* was not necessary to retrieve the most useful information from AF. The remaining experiments showed increasing *κ* as *L* became higher until the maximum was reached. In general, the optimum *κ* was reached combining intermediate values of values of *L* and *ν*, except for the AF + SpO_2_ subset. This setting reached the maximum *κ* with a large *L* and the lowest *ν*—Figure 5c. Nevertheless, differences between the maximum *κ* for different values of *ν* were not high.

### 4.5. Diagnostic Ability Assessment in the Test Set

Table 4 and Table 5 show the confusion matrices along with their respective *κ* and *Acc_4_* values obtained on the test set. Besides, the classification results of ODI 3% in the test set are shown in Table 6. Regarding multiclass classification, both *Acc_4_* and *κ* increased when features from both signals were combined. The highest performances were obtained when ODI 3% was also included. The highest overall *Acc_4_* and *κ* were achieved using the AF + SpO_2_ + ODI subset, although the same *Acc_4_* but slightly lower *κ* were reached using AF + ODI. It is important to note that AdaBoost models were more accurate than ODI 3%—Except for the AF model.

Table 7 shows the diagnostic performance in the test set for each setting in terms of their ability to predict the presence of OSA using the reference AHI cutoffs. Despite the lower *κ* in the four-class classification task, the AF + ODI subset reached the maximum *Acc* in all AHI cutoffs: *Acc* = 81.28% (Se = 92.06%; Sp = 36.00%), *Acc* = 82.05% (Se = 76.03%; Sp = 85.66%), and *Acc* = 90.26% (Se = 62.65%; Sp = 97.72%) in 1, 5 and 10 e/h, respectively. These results were the same for the AF + SpO_2_ + ODI subset in 5 e/h and 10 e/h, but it reached lower diagnostic performance in 1 e/h. Therefore, the AF + ODI subset showed the highest diagnostic ability in all AHI cutoffs, outperforming the SpO_2_ + ODI and AF + SpO_2_ + ODI subset in 1 e/h. Nevertheless, the SpO_2_ + ODI model also reached high diagnostic performance. 

## 5. Discussion

This study aimed to assess AF and SpO_2_ signals in the context of pediatric OSA and to evaluate whether these signals can provide complementary information to predict OSA severity in children. Furthermore, the diagnostic ability of multiclass AdaBoost classifiers was evaluated using six different combinations of features extracted from AF and SpO_2_ signals. Feature selection revealed that the relevant features from each signal remained non-redundant when both signals were combined, thus suggesting their complementarity. Moreover, the diagnostic ability increased when both signals were combined. Two novel contributions have been introduced in this paper. First, we have compared the diagnostic ability of the automatic signal processing of AF and SpO_2_ signals in the context of pediatric OSA. Second, we have designed and validated multiclass AdaBoost classifiers to predict the severity of OSA in children. To the best of our knowledge, this is the first time that AF and SpO_2_ signals are jointly evaluated in the context of pediatric OSA detection.

### 5.1. Feature Extraction and Selection

We characterized AF and SpO_2_ signals using time-domain statistics, nonlinear measures and spectral analysis. We defined a BOI in the AF signal between 0.134–0.176 Hz. Previous studies also focused on the analysis of specific BOIs in the context of pediatric OSA. Gutiérrez-Tobal et al. found two spectral BOIs (0.119–0.192 Hz and 0.784–0.890 Hz) using an AHI cutoff of 3 e/h [30]. In our study, however, 1, 5 and 10 e/h cutoffs were used. Our BOI is consistent with the first BOI defined in that work [30] and may be related to the presence of apneic events. Intermittent disruptions of at least two cycles in the normal respiratory flow define apneas and hypopneas and can increase the power in frequencies around and below one half of the normal respiratory frequency. Both BOIs are centered in 0.155 Hz, which is approximately half of the central frequency of the normal respiratory band in children [30]. In contrast, no significant differences between severity groups were found in the PSDs in higher frequencies. This might be due to the use of three different AHI cutoffs and the analysis of a larger cohort.

Previous studies focused on the spectral analysis of AF signals in the context of pediatric OSA found relevant features from their respective BOIs [30,33]. In our study, features from the BOI were discarded in the feature selection stage due to redundancy. CTM_AF_ and SpecEn^2^_AF_ were the most relevant and complementary AF features while time domain statistical moments were found redundant. Previous studies addressing irregularity and variability of AF signals in the context of pediatric OSA reported the positive association of CTM and SpecEn with OSA severity [31]. In this study, CTM_AF_ and SpecEn^2^_AF_ were found relevant and nonredundant among AF features, thus reinforcing previous findings.

The correlations with the AHI were higher in SpO_2_-derived features in comparison with AF-derived ones, suggesting that features from the SpO_2_ signal were more relevant. However, the majority of SpO_2_ features were removed due to redundancy with ODI 3%. Only M4F_SpO2_ was found non-redundant with CTM_SpO2_ and ODI 3%. These results confirm that the most useful information of SpO_2_ to detect OSA is summarized in ODI 3%. This finding is also supported in previous studies. Hornero et al. [26] assessed a similar set of features from SpO_2_ recordings, resulting in ODI 3% and M3F_SpO2_ being selected. Besides, Vaquerizo-Villar et al. [29] found one SpO_2_-derived feature from Detrended Fluctuation Analysis complementary with ODI 3%.

A novel contribution of this study is the joint assessment of AF and SpO_2_ signals using signal processing algorithms. It is remarkable that features from both signals were selected when AF and SpO_2_ features were combined. However, two different situations need to be analyzed. When feature selection was conducted on the AF + SpO_2_ set, selected features matched the features selected from AF and SpO_2_ sets separately. These features were thus non-redundant and may indicate complementarity between both signals. Conversely, SpecEn^2^_AF_ and CTM_SpO2_ were found redundant in settings with ODI 3%. Overall results of feature selection suggest that the information of AF and SpO_2_ signals could be complementary. These findings are in accordance with previous studies combining AF-derived features with ODI 3%, which reported not only their complementarity, but also an increase in the diagnostic performance when used together [30,32]. Accordingly, the complementarity of the information from AF and SpO_2_ signals in the context of adult OSA [34] is also confirmed in this study using a pediatric population.

### 5.2. Diagnostic Ability and Comparison with Previous Studies

In this study, novel multiclass AdaBoost classifiers have been introduced to predict OSA severity in children. The highest four-class accuracies were reached using the AF + ODI and the AF + SpO_2_ + ODI subsets. These results, together with the low accuracies reached using AF, SpO_2_ and ODI 3% separately, suggest that AdaBoost was able to take advantage of the information of AF and SpO_2_ signals. Moreover, the most useful information of the SpO_2_ seems to be summarized in ODI 3%. It is necessary to note that AF + SpO_2_ + ODI reached the highest *κ* but AF + ODI obtained the same *Acc_4_*. This slight difference can be related to the calculation of *κ*, that gives more importance to class imbalance [47]. The AF + SpO_2_ + ODI setting was slightly more accurate than AF + ODI classifying actual No OSA and Moderate OSA subjects, which were the least represented groups in our database. Thus, *κ* was higher in the AF + SpO_2_ + ODI subset. Both *Acc_4_* and *κ* were slightly lower using SpO_2_ + ODI, showing that oximetry alone can also achieve high diagnostic ability by means of AdaBoost. Nevertheless, the number of Moderate and Severe OSA subjects misclassified as No OSA was lower using AF + ODI. Another difference between these settings was observed in the number of overestimated subjects (the predicted severity of OSA was higher than the actual severity), which was also higher using SpO_2_ + ODI. The rates of underestimated and overestimated subjects were the most balanced in the AF + SpO_2_ + ODI setting: 20.77% and 21.28%, respectively. Using ODI 3% only, 40.26% and 14.62% of the subjects were underestimated and overestimated, respectively. Previous studies reported that ODI 3% alone underestimates the severity of OSA [29,32]. In this study, this tendency was also observed. On the other hand, the MLP neural networks used in previous approaches tended to overestimate the severity of OSA [29,32,42]. Vaquerizo-Villar et al. reported 12.75% of underestimated subjects and 27.30% of overestimated patients [29], while in Xu et al. the rates of underestimated and overestimated severity were 15.05% and 31.25%, respectively [42]. In our study, this behavior was not observed, since AdaBoost achieved a more balanced ratio of underestimated and overestimated subjects. These AdaBoost models were aimed at predicting OSA in a pediatric population. PSG data from boys and girls up to 13 years old was equally distributed in training and test sets. In general, no significant differences (*p* > 0.01) were found in age, sex or BMI *z*-score between patients correctly and incorrectly classified. We only found some differences in age and BMI *z*-score between rightly and incorrectly classified patients, which were limited to Mild OSA patients. Overall, diagnostic performances seem not to be biased towards any specific age, sex or BMI subgroup.

Regarding the results of binary classification, the top performing subset in 1 e/h was AF + ODI, reaching the highest Acc and NPV as well as the lowest LR-. These results suggest that AF + ODI is more suitable to discard the presence of OSA in 1 e/h since it was able to reduce false negatives. In comparison with AF + SpO_2_ + ODI, Se was higher and Sp slightly lower in AF + ODI. Nevertheless, differences were not high. AF + ODI and AF + SpO_2_ + ODI obtained the same diagnostic performance in 5 and 10 e/h. These settings obtained the highest Acc and the most balanced PPV and NPV in both cutoffs. Moreover, the value of LR+ in 10 e/h is remarkable since it indicates a very high likelihood when the AdaBoost model predicts a subject as Severe OSA. The differences between AF + ODI, SpO_2_ + ODI and AF + SpO_2_ + ODI were not high, which might suggest that the benefits of including AF are minor. Nevertheless, the diagnostic performance of models combining ODI 3% and AF reflects the complementarity of both signals. The contribution of AF reduced the number of false positives in 1 e/h using AF + ODI, which may compensate the added complexity and inconvenience of recording AF in children. Previous studies have successfully evaluated the usefulness of simplified devices to detect pediatric OSA using AF and SpO_2_ [10,11]. To the best of our knowledge, this is the first study that jointly evaluates the diagnostic ability of AF and SpO_2_ signals in children using signal processing methods. It would be convenient to enhance the diagnostic ability of these signals using signal processing methods alternative to those used in this study [29,41].

Table 8 summarizes the results achieved in previous studies focused on OSA detection in children. Simple and widespread binary classifiers (e.g., LR, LDA) were assessed in shorter cohorts, while MLP neural networks were proposed in studies comprising a larger number of subjects and using holdout validation (i.e., training and test sets). Most of the studies employed the 5 e/h AHI cutoff for binary classification, with Acc in the range 76.0–86.6%. Our proposal reached the highest Acc in 5 e/h among approaches using three AHI cutoffs. Moreover, it was close to the highest Acc among binary classifiers. In this study, Sp in 5 e/h was close to the highest ones in comparison with both binary and multiclass approaches, while Se was similar to those reached by MLP-based methods. Nevertheless, methods with higher Sp also exhibited lower Se. Fewer studies assessed their diagnostic ability in 1 e/h and 10 e/h cutoffs. Some of them developed independent binary LR models for each cutoff, while others relied on MLP neural networks. The former group reached more balanced Se-Sp pairs in both cutoffs, while the latter achieved higher Acc. The AdaBoost classifiers evaluated in this study also reached high Acc in all cutoffs. Other MLP-based approaches tended to overestimate OSA severity, resulting in low Sp in 1 e/h [29,32,42]. Our multiclass AdaBoost classifier achieved a higher Sp while maintaining high Se in 1 e/h using AF + ODI. On the other hand, Acc in 1 e/h was close to those reached using MLP networks. Therefore, a smaller proportion of symptomatic children without polysomnographically diagnosed OSA would be incorrectly diagnosed as suffering from OSA in comparison with other studies. Overall, the results of this study suggest that our ensemble learning-based approach succeeded in achieving high diagnostic ability. The performance of our AdaBoost-based approach strengthens the usefulness of ensemble learning as a valid alternative to other machine learning algorithms.

### 5.3. Limitations and Future Work

In spite of the promising performance of our proposal, some limitations and future investigations have to be pointed out. The database employed in this study comprised 974 subjects. Although this cohort is large, all subjects were recruited in the same center. It would be desirable to expand our database including new subjects from different sleep laboratories to further generalize our results. Secondly, we successfully evaluated AF and SpO_2_ signals separately and jointly in the context of childhood OSA detection. Future investigations may rely on potential incorporation of other useful signals included in the PSG. In this sense, the AF signals employed in this study were recorded using a thermistor. Comparison between nasal pressure sensor and thermistor AF signals would also constitute a future goal. In addition, the AF-derived inter breath interval series can be considered for future studies to enhance the diagnostic ability of AF. Signals have been characterized using widespread signal processing methods in the context of OSA. Future work may comprise alternative approaches like bispectrum and wavelets, as well as other nonlinear analyses. Finally, although AdaBoost classifiers yielded high diagnostic performance, other ensemble learning methodologies like bagging or stacking can also be assessed to compare their diagnostic performance using SpO_2_ and AF signals.

## 6. Conclusions

The results of this study showed the usefulness of the joint analysis of AF and SpO_2_ signals in the context of pediatric OSA. A remarkable diagnostic performance was achieved using a multiclass AdaBoost classifier fed with a combination of relevant and complementary information from both signals. The most accurate AdaBoost model successfully combined CTM_AF_ with ODI 3%, which was found the most useful parameter of the SpO_2_ signal. This joint model outperformed the diagnostic ability of each of these signals separately. Furthermore, we derived an accurate and unbiased AdaBoost model able to decrease the underestimation of the OSA severity previously observed in ODI 3%. Our dual-channel approach is thus a potential alternative to single-channel methodologies, one that might be useful to deploy in the context of simplified screening methods aimed at detecting OSA in children.

## Figures and Tables

**Table 1:** Sociodemographic and clinical data of the subjects involved in the study. Subjects distributions represented as N° (%). Age, normalized body mass index (BMI *z*-score) and apnea–hypopnea index (AHI) represented as the median (interquartile range).

	All	Training Set	Test Set
N° of Subjects	974	584 (60%)	390 (40%)
Age (years)	6.00 [3.00, 8.00]	6.00 [3.00, 8.00]	5.50 [3.00, 9.00]
N° of Males	599 (61.50%)	346 (59.25%)	253 (64.87%)
N° of Females	375 (38.50%)	238 (40.75%)	137 (35.13%)
BMI *z-*score	−0.22 [−0.60, 0.37]	−0.24 [−0.61, 0.43]	−0.17 [−0.58, 0.27]
AHI (events/hour)	3.80 [1.53, 9.35]	4.08 [1.71, 10.00]	3.30 [1.40, 7.87]
N° of No OSA	171 (17.56%)	96 (16.44%)	75 (19.23%)
N° of Mild OSA	398 (40.86%)	229 (39.21%)	169 (43.33%)
N° of Moderate OSA	176 (18.07%)	113 (19.35%)	63 (16.15%)
N° of Severe OSA	229 (23.51%)	146 (25.00%)	83 (21.28%)

Normalized body mass index (BMI *z*-score); apnea–hypopnea index (AHI); Obstructive Sleep Apnea (OSA).

**Table 2:** Spearman’s correlation coefficient of sample entropy with apnea–hypopnea index in the training set for different values of the parameters *m* and *r*.

	AF			SpO_2_		
	*m* = 1	*m* = 2	*m* = 3	*m* = 1	*m* = 2	*m* = 3
*r* = 0.05	−0.0872	**−0.1187**	0.0026	0.5502	0.5516	**0.5586**
*r* = 0.10	−0.0753	−0.0863	−0.1168	0.5123	0.5118	0.5134
*r* = 0.15	−0.0777	−0.0802	−0.0914	0.4786	0.4786	0.4784
*r* = 0.20	−0.0832	−0.0824	−0.0886	0.4395	0.4381	0.4399
*r* = 0.25	0.0897	−0.0880	−0.0910	0.3895	0.3899	0.3900
*r* = 0.30	0.0983	−0.0951	−0.0966	0.3341	0.3350	0.3367

Airflow (AF) signal; (b) oximetry (SpO_2_). Maximum absolute values represented in bold.

**Table 3:** Spearman’s correlation coefficients (*ρ*) with the apnea–hypopnea index and their corresponding *p*-values of features in the training set and *p*-values of the Kruskal–Wallis test.

Feature	AF			SpO_2_		
	Spearman		Kruskal–Wallis	Spearman		Kruskal–Wallis
	*ρ*	*p*-Value	*p*-Value	*ρ*	*p*-Value	*p*-Value
*M1T*	0.1693	<<0.01	0.0061 *	−0.4135	<<0.01	<<0.01
*M2T*	−0.2481	<<0.01	<<0.01	0.5145	<<0.01	<<0.01
*M3T*	−0.1655	<<0.01	0.0024 *	−0.1879	<<0.01	<<0.01
*M4T*	0.3580	<<0.01	<<0.01	0.0968	0.0194	0.0103 *
*MedT*	0.2070	<<0.01	<<0.01	−0.3467	<<0.01	<<0.01
*CTM*	0.3979	<<0.01	<<0.01	−0.6187	<<0.01	<<0.01
*LZC*	−0.0660	0.1111	0.0409 *	0.3871	<<0.01	<<0.01
*SampEn*	−0.1187	<0.01	0.0270 *	0.5586	<<0.01	<<0.01
*M1F*	0.3492	<<0.01	<<0.01	0.6773	<<0.01	<<0.01
*M2F*	0.2979	<<0.01	<<0.01	0.6352	<<0.01	<<0.01
*M3F*	−0.1418	<<0.01	<<0.01	0.0184	0.6574	0.4893 *
*M4F*	−0.0967	0.0195	0.0112*	0.0356	0.3899	0.4643 *
*MedF*	0.3591	<<0.01	<<0.01	0.6753	<<0.01	<<0.01
*MaxF*	0.3245	<<0.01	<<0.01	0.6646	<<0.01	<<0.01
*MinF*	0.3588	<<0.01	<<0.01	0.6504	<<0.01	<<0.01
*FreqM*	−0.1280	<0.01	0.0117 *	0.1209	<0.01	0.0073 *
*SpecEn*	0.3464	<<0.01	<<0.01	0.0060	0.8842	0.9340 *
*SpecEn* ^2^	0.2741	<<0.01	<<0.01	0.1247	<0.01	0.0234 *
*SpecEn* ^3^	0.1304	<0.01	0.0024 *	0.1075	<0.01	0.0742 *
ODI 3%	—	—	—	0.6918	<<0.01	<<0.01

*: Not lower than the Bonferroni corrected *p*-value (*p* = 0.01/6). Airflow (AF) signal; (b) oximetry (SpO_2_).

**Table 4:** Confusion matrices of the predictions of AdaBoost models in the test set using the subsets AF, SpO_2_ and AF + SpO_2_.

		AdaBoost (Without ODI 3%)											
Severity Levels		Estimated: AF				Estimated: SpO_2_				Estimated: AF + SpO_2_			
		No	Mild	Mod.	Sev.	No	Mild	Mod.	Sev.	No	Mild.	Mod.	Sev.
Actual	No	1	55	16	3	17	50	8	0	19	47	8	1
	Mild	1	97	53	18	19	119	30	1	21	111	35	2
	Mod.	1	29	22	11	5	29	24	5	6	24	27	6
	Sev.	0	25	25	33	3	12	34	34	2	8	36	37
		*Acc_4_* = 39.23%; *κ* = 0.1143				*Acc_4_* = 49.74%; *κ* = 0.2646				*Acc_4_* = 49.74%; *κ* = 0.2781			

**Table 5:** Confusion matrices of the predictions of AdaBoost models in the test set using the subsets AF + ODI, SpO_2_ + ODI and AF + SpO_2_ + ODI.

		AdaBoost (With ODI 3%)											
Severity Levels		Estimated: AF + ODI				Estimated: SpO_2_ + ODI				Estimated: AF + SpO_2_ + ODI			
		No	Mild	Mod.	Sev.	No	Mild	Mod.	Sev.	No	Mild	Mod.	Sev.
Actual	No	27	44	3	1	26	45	3	1	28	43	3	1
	Mild	23	115	30	1	23	113	32	1	25	113	30	1
	Mod.	2	24	32	5	7	18	32	6	7	18	33	5
	Sev.	0	9	22	52	1	8	22	52	2	8	21	52
		*Acc_4_* = 57.95%; *κ* = 0.3930				*Acc_4_* = 57.18%; *κ* = 0.3864				*Acc_4_* = 57.95%; *κ* = 0.3984			

**Table 6:** Confusion matrix of the predictions of ODI 3% in the test set.

		ODI 3%			
Severity Levels		Estimated			
		No	Mild	Mod.	Sev.
Actual	No	65	7	1	2
	Mild	110	35	11	13
	Mod.	18	14	8	23
	Sev.	6	6	3	68
		*Acc_4_* = 45.13%; *κ* = 0.2833			

**Table 7:** Diagnostic performances of AdaBoost models and ODI 3% in the test set in the apnea–hypopnea index cutoffs 1, 5 and 10 events/hour (e/h).

Cutoff	Subset	Se	Sp	Acc	PPV	NPV	LR+	LR-
1 e/h	AF	99.37%	1.33%	80.51%	80.88%	33.33%	1.0071	0.4762
	SpO_2_	91.43%	22.67%	78.21%	83.24%	38.64%	1.1823	0.3782
	AF + SpO_2_	90.79%	25.33%	78.21%	83.63%	39.58%	1.2160	0.3634
	AF + ODI	92.06%	36.00%	81.28%	85.80%	51.92%	1.4385	0.2205
	SpO_2_ + ODI	90.16%	34.67%	79.49%	85.29%	45.61%	1.3800	0.2839
	AF + SpO_2_ + ODI	89.21%	37.33%	79.23%	85.67%	45.16%	1.4235	0.2891
	ODI 3%	57.46%	86.67%	63.08%	94.76%	32.66%	4.3095	0.4908
5 e/h	AF	62.33%	63.11%	62.82%	50.28%	73.68%	1.6898	0.5969
	SpO_2_	66.44%	84.02%	77.44%	71.32%	80.71%	4.1567	0.3995
	AF + SpO_2_	72.60%	81.15%	77.95%	69.74%	83.19%	3.8511	0.3376
	AF + ODI	76.03%	85.66%	82.05%	76.03%	85.66%	5.3002	0.2799
	SpO_2_ + ODI	76.71%	84.84%	81.79%	75.17%	85.89%	5.0589	0.2745
	AF + SpO_2_ + ODI	76.03%	85.66%	82.05%	76.03%	85.66%	5.3002	0.2799
	ODI 3%	69.86%	88.93%	81.79%	79.07%	83.14%	6.3135	0.3389
10 e/h	AF	39.76%	89.58%	78.97%	50.77%	84.62%	3.8144	0.6725
	SpO_2_	40.96%	98.05%	85.90%	85.00%	86.00%	20.9598	0.6021
	AF + SpO_2_	44.58%	97.07%	85.90%	80.43%	86.63%	15.2062	0.5710
	AF + ODI	62.65%	97.72%	90.26%	88.14%	90.63%	27.4768	0.3822
	SpO_2_ + ODI	62.65%	97.39%	90.00%	86.67%	90.61%	24.0422	0.3835
	AF + SpO_2_ + ODI	62.65%	97.72%	90.26%	88.14%	90.63%	27.4768	0.3822
	ODI 3%	81.93%	87.62%	86.41%	64.15%	94.72%	6.6189	0.2063

**Table 8:** Diagnostic performances of state-of-the-art approaches in the context of childhood Obstructive Sleep Apnea syndrome.

Study	N	Signal	Methods (Extraction/Selection/Classification)	Validation	Cutoff	Se	Sp	Acc
Chang et al. (2013) [36]	141	SpO_2_	ODI, questionnaires/-/LR	--	5	60.0	86.0	76.6
Wu et al. (2017) [37]	311	—	Clinical parameters/-/Stepwise LR	Holdout	5	94.8	25.0	78.2
Gil et al. (2010) [38]	21	PPG	DAP events, HRV, PTTV/Wrapper/LDA	--	5	75.0	85.7	80.0
Lázaro et al. (2014) [39]	21	PPG	DAP events, spectral analysis of PRV/Wrapper/LDA	--	5	100	71.4	86.6
Garde et al. (2014) [23]	146	SpO_2_, PRV	Time, frequency, nonlinear/-/LDA	Four-fold	5	88.4	83.6	84.9
Garde et al. (2019) [24]	207	SpO_2_, PRV	Time, frequency, ODI (SpO_2_); standard spectral bands (PRV)/-/LR (3 binary models)	Holdout	1	68.0	86.0	71.0
					5	58.0	89.0	78.0
					10	90.0	87.0	88.0
Álvarez et al. (2018) [28]	142	SpO_2_	Time domain, ODI, symbolic dynamics/FSLR/LR	Bootstrap	5	73.5	89.5	83.3
Barroso-Garcia et al. (2017) [31]	501	AF	CTM and SpecEn/FSLR/LR (3 binary models)	Holdout	1	60.5	58.6	60.0
					5	65.0	80.6	76.0
					10	83.3	79.0	80.0
Crespo et al. (2018) [40]	176	SpO_2_	Time, frequency, nonlinear, ODI/FCBF/LDA, QDA, LR (3 binary models)	Bootstrap	1	93.9	37.8	84.3
					5	70.0	91.4	82.7
Hornero et al. (2017) [26]	4191	SpO_2_	Time, frequency, nonlinear, ODI/FCBF/MLP regression	Holdout	1	84.0	53.2	75.2
					5	68.2	87.2	81.7
					10	68.7	94.1	90.2
Xu et al. (2019) [42]	432	SpO_2_	ODI, M3F/-/MLP regression	Direct validation	1	95.3	19.1	79.6
					5	77.8	80.5	79.4
					10	73.5	92.7	88.2
Vaquerizo-Villar et al. (2018) [29]	981	SpO_2_	DFA, ODI/FCBF/MLP regression	Holdout	1	97.1	23.3	82.7
					5	78.8	83.7	81.9
					10	77.1	94.8	91.1
Barroso-García et al. (2020) [32]	946	AF, ODI	Recurrence plots, ODI/FCBF/Bayesian MLP regression	Holdout	1	97.7	22.2	83.2
					5	78.7	78.3	78.5
					10	78.8	94.3	91.0
This Study	974	AF, SpO_2_	Time, Frequency, Nonlinear, ODI/FCBF/Multiclass AdaBoost	Holdout	1	92.1	36.0	81.3
					5	76.0	85.7	82.1
					10	62.7	97.7	90.3

Airflow signal (AF); Central Tendency Measure (CTM); Decreases in Amplitude of Plethysmography (DAP); Detrended Fluctuation Analysis (DFA); Fast Correlation-Based Filter (FCBF); Forward Stepwise Logistic Regression (FSLR); Heart Rate Variability (HRV); Linear Discriminant Analysis (LDA); Logistic Regression (LR); third order moment in frequency domain (M3F); Multilayer Perceptron (MLP); number of subjects (N); Oxygen Desaturation Index (ODI); Photoplethysmography (PPG); Pulse Rate Variability (PRV); Pulse Transit Time Variability (PTTV); Quadratic Discriminant Analysis (QDA); Spectral Entropy (SpecEn); oxygen saturation signal (SpO_2_).
